# Metabolic and Lipoprotein Changes in Type 2 Diabetes Patients Treated with Empagliflozin

**DOI:** 10.3390/biomedicines14092016

**Published:** 2026-09-08

**Authors:** Evagelia E. Habeos, Georgios K. Markantes, Anastasia Parthymou, Konstantinos Dimitropoulos, George I. Habeos, Marios Papasotiriou, Markos Marangos, Dionysios V. Chartoumpekis

**Affiliations:** 1 Division of Endocrinology, Department of Internal Medicine, School of Medicine, University of Patras, 26504 Patras, Greecenatassap@yahoo.com (A.P.);; 2 Department of Nephrology and Kidney Transplantation, University Hospital of Patras, 26504 Patras, Greece; 3 Division of Infectious Diseases, Department of Internal Medicine, University of Patras, 26504 Patras, Greece

**Keywords:** SGLT2, SGLT2 inhibitors, inflammation, lipids, iron metabolism

## Abstract

**Background/Objectives:** This study evaluated the metabolic effects of empagliflozin, an SGLT2 inhibitor (SGLT2i), in type 2 diabetes patients. **Methods:** In an observational cohort study, 42 patients received empagliflozin for 1 month, with serum and urine analyzed for IL-6, NT-proBNP, iron metabolism markers, and electrolytes. Another group was treated for 3 months, assessing lipoprotein and glycoprotein profiles, IL-6, and NT-proBNP. **Results:** Empagliflozin increased serum magnesium (mean difference = 0.26 ± 0.38 mg/dL, *p* < 0.0001), hemoglobin (mean difference = 0.45 ± 0.78 g/dL, *p* = 0.0005), and hematocrit (mean difference = 1.74 ± 2.08%, *p* < 0.0001), while decreasing ferritin (mean difference = −29.23 ± 51.97 ng/mL) and transferrin saturation (mean difference = −3.65 ± 7.22%). NT-proBNP and IL-6 levels trended lower (non-significant). Lipoprotein profiling showed a neutral effect. **Conclusions:** Empagliflozin positively affected serum magnesium, iron metabolism, and inflammatory markers without altering lipoprotein profiles. Further research is needed to elucidate SGLT2i’s molecular mechanisms underlying their pleiotropic–cardioprotective effects.

## 1. Introduction

It is well known that obesity and type 2 diabetes mellitus (T2DM) have reached epidemic proportions [1,2], and the use of treatments with pleiotropic actions that are not limited to the glucose-lowering effects is highly needed. The use of sodium–glucose cotransporter 2 (SGLT2) inhibitors (SGLT2is) is increasing, as they reduce the risk of cardiovascular death and hospitalizations for heart failure in patients with T2DM, chronic kidney disease (CKD), or heart failure with a reduced (HFrEF) or preserved ejection fraction (HFpEF) [3,4]. This benefit might be related to enhanced nutrient deprivation signaling pathways in cardiomyocytes and consequently decreased cellular stress in a variety of conditions such as hyperglycemia, ischemia or pressure overload [5]. In this context, the effects of SGLT2is on iron metabolism have been investigated. Anemia is a common finding in patients with T2DM, especially in those with albuminuria and renal failure [6,7,8,9]. Several randomized clinical studies that explored the effects of SGLT2is in diabetic patients with or without CKD showed that the hemoglobin levels increased after treatment with SGLT2is [2,10,11,12,13,14,15,16,17,18]. However, these studies differ substantially in the groups of patients recruited. Specifically, most of the studies included patients with chronic kidney disease [10,11,12,16,18]; none of them recruited specifically patients with heart failure. Maruyama et al. included patients with anemia [16], and Oshima et al. analyzed both patients with and without anemia [18]. It is true that in these studies the shift of hemoglobin is modest (0.5–0.9 g/dL); hence, the clinical significance is not well established. Regarding iron parameters, although there were no significant changes in TSAT, serum ferritin levels were decreased (*p*  =  0.003) in the study of Maruyama et al. [16]. In a post hoc analysis of the CREDENCE trial it was found that iron deficiency was highly prevalent in patients with T2D and chronic kidney disease, and canagliflozin increased TIBC and decreased ferritin [19]. The increase in hemoglobin/hematocrit was initially attributed primarily to hemoconcentration resulting from the diuretic action of SGLT2i [17]. However, this explanation does not completely account for the phenomenon, as a transition from a diuretic to an SGLT2 inhibitor is characterized by an increase in hematocrit [20,21]. Thus, an increase in erythropoietin production has been proposed as a mechanism. There are already some studies supporting this possibility in diabetic patients. Specifically, Maruyama et al. reported an increase by 38% of EPO [16], while Mazer et al. reported an adjusted between-groups difference of 3.86 mIU/mL at 1 month of treatment and 1.91 mIU/mL at 6 months, with the latter one only being statistically significant [22]. A transient increase in EPO at 6 weeks of treatment was also reported in the RCT by Ghanim et al. [23].

Moreover, the cardiovascular outcome trials of SGLT2is showed a lower risk of hospitalization for heart failure, a lower risk of major cardiovascular events, and a lower risk of all-cause mortality in individuals with diabetes [24] as well as in individuals with heart failure without diabetes [4]. However, the exact mechanism behind these beneficial cardiovascular effects is largely unclear. Since low-density lipoproteins (LDLs) and triglyceride-rich lipoproteins are known as causal risk factors for atherosclerotic cardiovascular disease [25], the effect of SGLT2 inhibition on lipoprotein levels is of great interest. However, there is a lack of systematic measuring and reporting of lipid and lipoprotein levels in randomized placebo-controlled trials of SGLT2is. In addition, SGLT2is increase total cholesterol, LDL cholesterol, and HDL cholesterol and lower triglycerides in several meta-analyses of randomized trials [26,27,28].

The aim of this study was to characterize the short-term effects of empagliflozin on erythropoiesis, iron metabolism, inflammatory markers, and lipoproteins in patients with type 2 diabetes mellitus naïve to SGLT2 inhibition.

## 2. Materials and Methods

**Figure 1:**
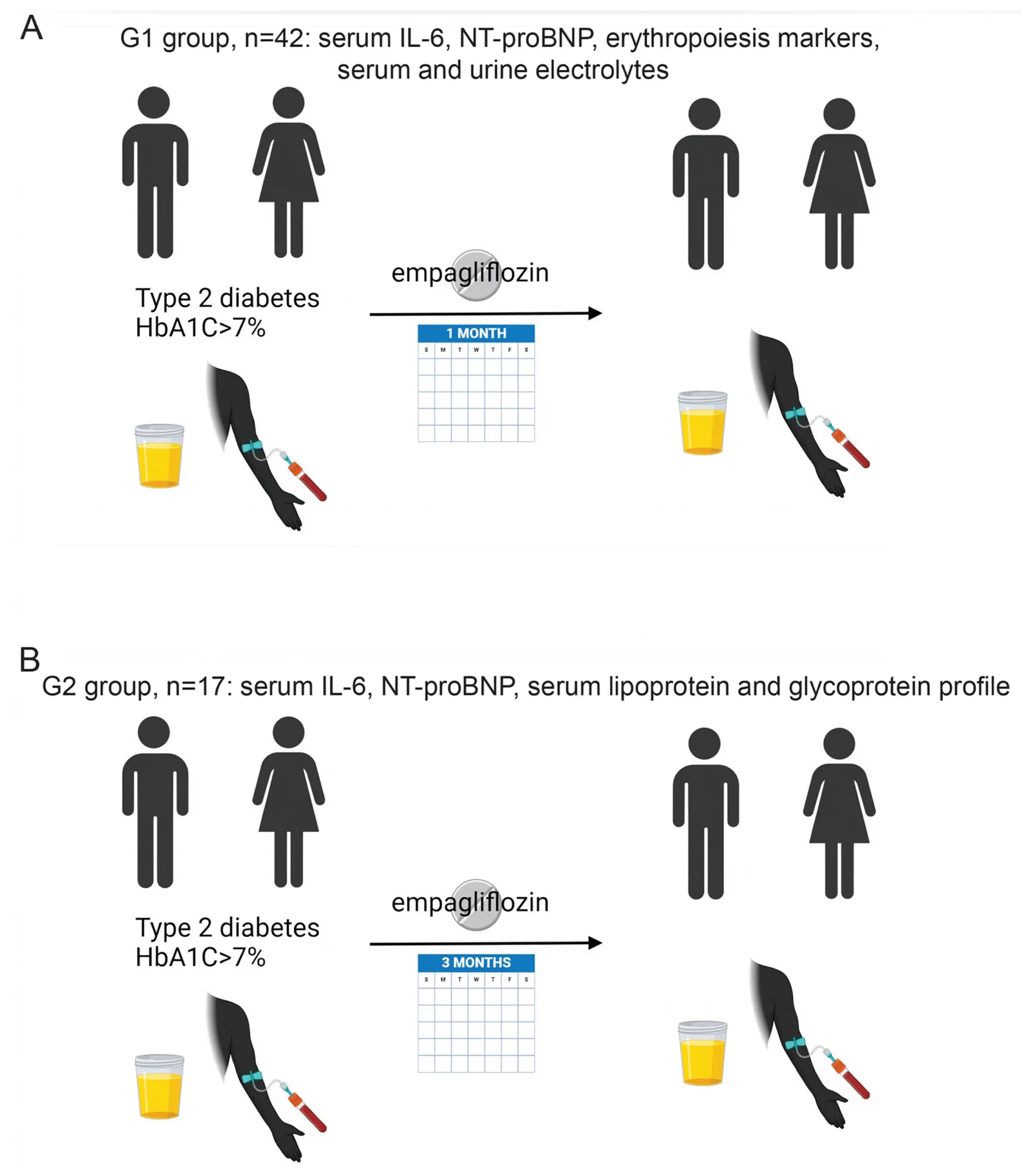
Clinical study design. (**A**) First group (G1) with *n* = 42 treated with empagliflozin for 1 month. (**B**) Second group (G2) with *n* = 17 patients treated with empagliflozin for 3 months. Created in BioRender. Chartoumpekis, D. (2026) https://BioRender.com/ycy0bdr (accessed on 1 July 2026).

This is an observational cohort study that was conducted at the Diabetes Center of the University Hospital of Patras, Greece (ClinicalTrials.gov NCT05408936). A total of 50 patients with T2DM were recruited who were not previously treated with SGLT2i. The inclusion criteria were T2DM patients not controlled on their current treatment based on HbA1C level (HbA1C > 7% was considered as the cutoff for not properly controlled diabetes). Exclusion criteria were renal disease with an eGFR < 30 mL/min/1.73 m^2^, a history of frequent urinary tract infections, a known history of malignancy, hepatitis or cirrhosis, treatment for anemia (e.g., iron supplementation or erythropoietin [EPO] treatment), and ongoing infection or use of diuretics so as to remove any possibility of possible hemoconcentration at baseline. The eGFR was calculated based on the Chronic Kidney Disease Epidemiology Collaboration equation. Treatment with 25 mg empagliflozin daily was added to their existing antidiabetic regimen. No other modification of the antidiabetic treatment was done during the study. All patients were receiving statins as part of their regular treatment, and their dose was not changed during the study. Fasting blood sampling was performed in all patients before the initiation of the treatment and after 1 month. The final analysis included 42 subjects with matched blood and urine samples before and after initiation of the treatment. Five patients did not show up in a timely manner for the sampling after the initiation of the treatment and 3 were dropouts. At each visit, patients were assessed clinically, and a urine spot was collected after an overnight fast. Figure 1A summarizes the study protocol for this group of patients (G1). Laboratory testing included hemoglobin, hematocrit, HbA1c, serum creatinine, sodium, potassium, magnesium, iron, total iron binding capacity, ferritin, serum EPO, IL-6, NT-proBNP, urine creatinine, urine sodium, potassium, glucose, chloride, and the urine anion gap.

A smaller independent group of patients (*n* = 20, G2) were recruited using the same criteria described above for group G1, but the sampling was performed at baseline and after 3 months of empagliflozin treatment (Figure 1B). One patient dropped out of this study and two did not show up in a timely manner for the sampling after the initiation of the treatment; thus, 17 matched serum samples were analyzed for lipoprotein–glycoprotein profiles, NT-proBNP and IL-6. The rationale of using a longer-term exposure to empagliflozin in the second group was that it empirically takes more than 1 month to have significant changes in lipoproteins after an intervention or treatment [29]. A separate group, G2, was used to measure lipoproteins, as the samples of G1 at baseline were already thawed for other purposes and the follow-up period of 3 months had also passed.

### 2.1. Measurements

Serum and urine samples were stored in a −80 °C freezer. IL-6 and NT-proBNP were measured in a Cobas 6000 E601 analyzer (Roche Diagnostics GmbH, Mannheim, Germany) using electrochemiluminescence (ECL) technology for immunoassay analysis. EPO was measured in an Immulite 2000 analyzer (Siemens, Munich, Germany) using the relevant immunoassay. The rest of the blood chemistries were measured in an Olympus AU640 (Biosfer Teslab, Hamburg, Germany) analyzer. For the advanced lipoprotein testing, frozen serum samples were shipped in dry ice to Biosfer Teslab (Tarragona, Spain). The advanced lipoprotein testing was performed by 1H-NMR spectroscopy based on the Liposcale test as described previously [30]. The glycoprotein profile was also assessed using the same methodology in the same lab as described previously [31]. Briefly, global plasma glycoproteins GlycA and GlycB that represent the amount of acetyl groups of protein-bound N-acetylglucosamine, N-acetylgalactosamine, and N-acetylneuraminic acid were assessed. Spectral regions were deconvoluted by a line-shape fitting procedure as previously described [32], yielding for each peak its area—proportional to the concentration of the corresponding N-acetyl groups—and its shape factor, defined as the ratio of peak height to peak width at half maximum (H/W). Whereas the area reflects glycoprotein abundance, the H/W ratio reflects the mobility and aggregation state of the glycan chains: during the acute-phase response these signals become taller and narrower, so that higher H/W values indicate a more pronounced systemic inflammatory state [33]. H/W ratios are dimensionless and expressed in arbitrary units; GlycA and GlycB concentrations are expressed in µmol/L.

### 2.2. Statistics

All measurements for each parameter were paired (comparison before and after treatment for each patient). A paired *t*-test was used to compare the differences before and after the treatment for the larger group of patients (G1), and a Wilcoxon matched-pairs signed rank test was used for the smaller group of patients (G2) with the lipoprotein/glycoprotein measurements. Prism 9.0 (GraphPad Software, Boston, MA, USA) was used for the statistical analyses and for the visualization of the data/graphs generations. Statistical significance was set at *p* < 0.05.

### 2.3. Ethics

This observational cohort study that was conducted at the Diabetes Center of the University Hospital of Patras, Greece (ClinicalTrials.gov NCT05408936), was approved by the University Hospital of Patras Ethics Committee, approval ID 73/22 February 2022). Participation was based on written informed consent, and the study was conducted in accordance with the Declaration of Helsinki.

## 3. Results

### 3.1. Baseline Characteristics of the Study Subjects

The demographic characteristics of the study subjects are described in the tables below for the 1-month (Table 1) and 3-month (Table 2) exposure to empagliflozin. It is of note that patients in both groups did not have proteinuria and the baseline characteristics are not significantly different between groups G1 and G2.

### 3.2. Effects of SGLT2i on Serum and Urine Electrolytes After 1 Month of Treatment

**Figure 2:**
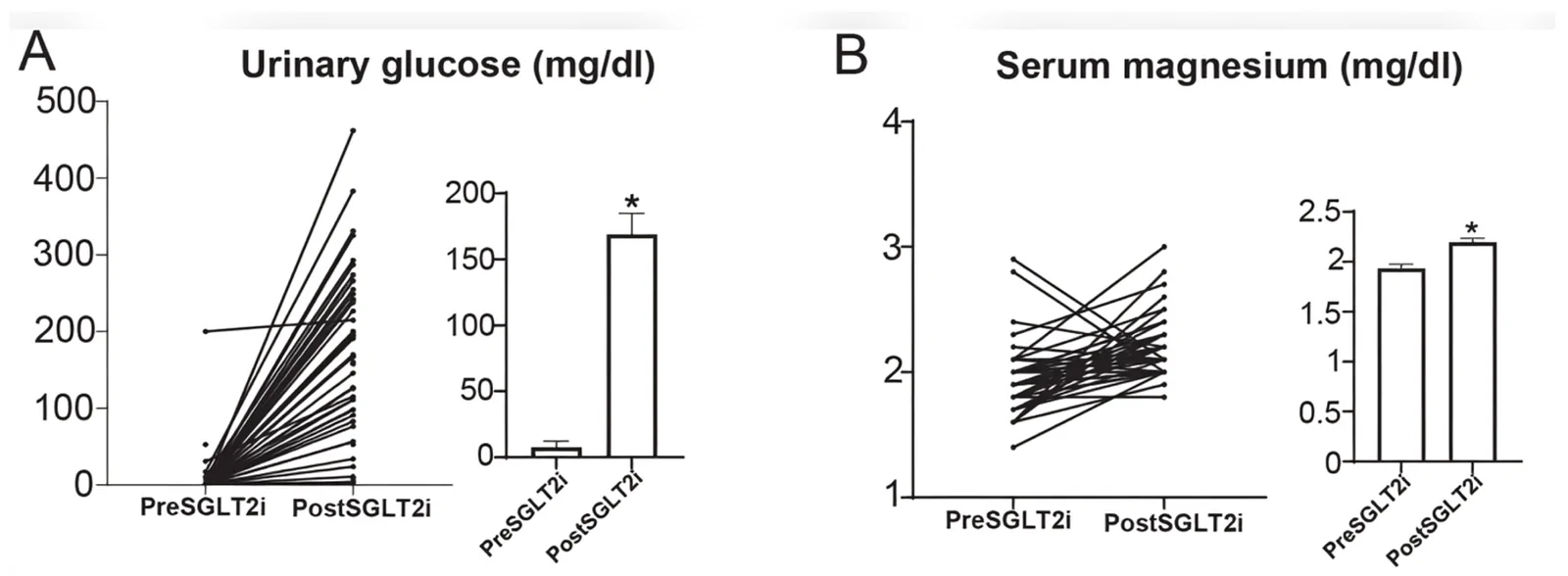
Urinary glucose levels (**A**) and serum magnesium levels (**B**) at baseline and after a 1-month treatment with empagliflozin. Bars show means ± SEM. * *p* < 0.05.

Urine glucose was measured so as to confirm that patients were compliant with the treatment. The SGLT2i increased glycosuria as expected (Figure 2A, Table 3). Serum potassium and sodium levels remained unchanged (Table 3), but magnesium levels were increased (mean difference 0.26 ± 0.38 mg/dL, *p* < 0.0001) (Figure 2B, Table 3). Serum creatinine was also increased (mean difference 0.076 ± 0.19 mg/dL, *p* = 0.0134, Table 3). The urine anion gap (Urinary [Na] + [K] − [Cl]) was measured as an indirect index of ammonium excretion, which reflects the net urinary acid excretion. The mild SGLT2i-induced ketonemia was expected to be detected as an increase in the urine anion gap. Nevertheless, no difference was detected in the urine anion gap after the treatment (Table 3).

### 3.3. Effects of SGLT2i on Iron Metabolism and Erythropoiesis

**Figure 3:**
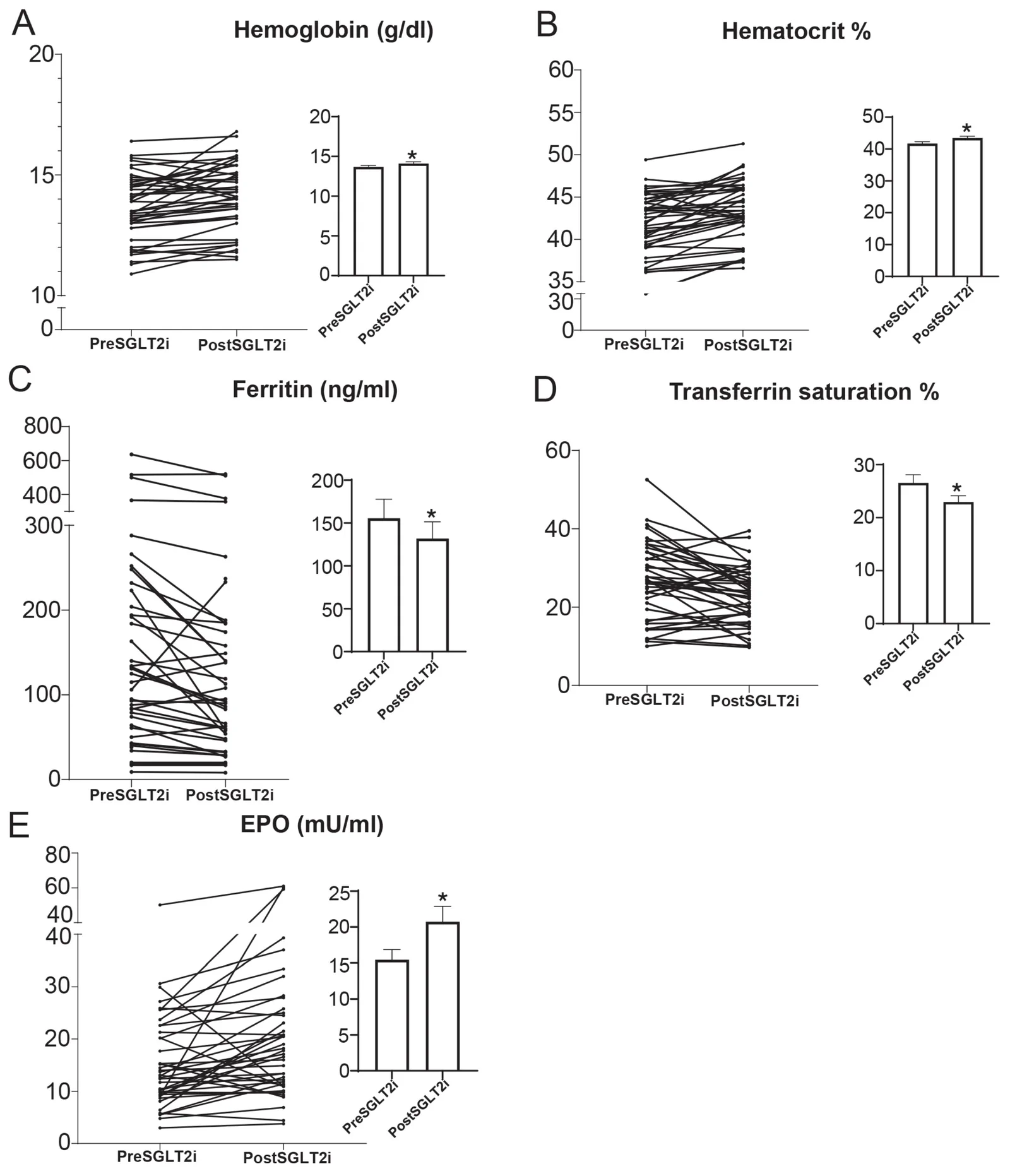
Serum levels of (**A**) hemoglobin, (**B**) hematocrit, (**C**) ferritin, (**D**) transferrin saturation and (**E**) erythropoietin (EPO) at baseline and after a 1-month treatment with empagliflozin. Bars show means ± SEM. * *p* < 0.05.

We sought to confirm previous reports regarding the effects of SGLT2is on hematocrit levels in this group of patients who were started on empagliflozin treatment as part of an add-on treatment for uncontrolled diabetes (HbA1C > 7%). SGLT2i treatment increased hemoglobin and hematocrit levels (Table 4). The mean difference in hemoglobin was 0.45 ± 0.78 g/dL (*p* = 0.0005) (Figure 3A) and in hematocrit was 1.74 ± 2.08% (*p* < 0.0001) (Figure 3B) after SGLT2i treatment. Ferritin levels were reduced from 158.6 ±142.3 to 129.2 ± 124.6 ng/mL (Figure 3C, Table 4). Iron levels remained unchanged after SGLT2i treatment, while TIBC was increased (mean difference = 25.48 ± 68.44, *p* = 0.0237) (Table 4), and consequently transferrin saturation was reduced (mean difference = −3.65 ± 7.22%) (Figure 3D, Table 4). Erythropoietin (EPO) was increased after SGLT2i treatment (mean difference = 5.28 ± 10.85, *p* = 0.0030) (Figure 3E, Table 4).

### 3.4. Effects of SGLT2i on Markers of Inflammation

One of the proposed mechanisms of cardiorenal protection of SGLT2is is their potential anti-inflammatory effect [34]. In addition, inflammation has an impact on iron metabolism [35]. For instance, during acute-phase inflammation, concentrations of the proteins ferritin and ceruloplasmin are elevated, while levels of transferrin are reduced [36]. In this study ferritin levels indeed decreased after the treatment (Table 4). Thus, in this context more markers of inflammation were evaluated (IL-6, NT-proBNP [37], GlycA, GlycB [38,39]). The levels of IL-6 were examined before and after SGLT2i treatment. No statistically significant difference was observed in IL-6 after SGLT2i treatment after one month (mean difference= −2.5 ± 16.04, *p* = 0.29) (Table 4) and three months of treatment (mean difference = −1.56 ± 9.08, *p* = 0.96) (Table 5). NT-proBNP also showed no significant difference after SGLT2i treatment both after one month (mean difference = −147.8 ± 895, *p* = 0.26) (Table 4) and three months of treatment (mean difference = −52.97 ± 125.2, *p* = 0.22) (Table 5). Last, the height/width ratios of GlycA and GlycB, an indirect marker of inflammation, was tested in patients treated for 3 months with an SGLT2i, but no difference was detected (Table 5).

### 3.5. Effects of SGLT2i on Cholesterol, Triglyceride and Particle Numbers in Individual Lipoprotein Classes

It is known that lipoproteins have an impact on atherosclerosis and cardiorenal diseases [40,41]. Although SGLT2is have a beneficial effect on atherosclerosis [42] and cardiovascular outcomes, their role on lipoprotein metabolism has not been thoroughly evaluated, and some studies indicate an increase in LDL cholesterol [28]. However, an in-depth evaluation of lipoprotein particles has not been performed. In this context, an NMR-based analysis of individual lipoprotein particles was performed before and after 3 months of treatment with an SGLT2i.

No difference was found in cholesterol or triglycerides in the individual lipoprotein classes (VLDL, LDL, IDL and HDL) (Table 5). In addition, no difference was found in individual lipoprotein particle numbers (large, medium, and small VLDL; large, medium and small LDL; large, medium and small HDL) (Table 5). The particle diameter of VLDL, LDL and HDL was similar before and after the treatment (Table 5). At the same time the concentration of glycoproteins GlycA, GlycB and GlycF, as well as the height/width ratios of GlycA and GlycB, were measured, and no difference was found with SGLT2i treatment (Table 5).

## 4. Discussion

Herein, we profiled T2D patients before and after the addition of empagliflozin in their regular treatment regarding its effects on hematopoiesis, electrolytes, markers of inflammation and lipoprotein profile.

In the present study an increase in magnesium concentrations was confirmed after SGLT2i treatment. A meta-analysis of 18 randomized controlled trials corroborated this effect of SGLT2is on T2D patients [43], which might be the effect of osmotic diuresis, but the exact mechanism and its physiological significance remain unknown. Nevertheless, this increase in magnesium concentration has been proposed as one of the favorable effects of SGLT2is to reduce cardiovascular mortality [44]. Regarding the urine anion gap, no changes were detected with SGLT2i treatment. The urine anion gap was proven to be a very rough measure of ammonium production, as it was insufficiently sensitive in detecting small changes that could be accounted for by daily dietary acid production. Creatinine increased as expected after one-month treatment with an SGLT2i.

SGLT2i treatment led to increased hematocrit and hemoglobin levels, as described previously [45], as well as to increased EPO levels. It is interesting that this effect is seen after just 1 month of treatment. Hemoglobin elevation serves as a statistical mediator of the potential of SGLT2is to reduce the risk of cardiovascular death and of hospitalizations due to heart failure in clinical trials [17,46]. It appears that this increase in hemoglobin is not related to hemoconcentration, as happens with diuretics, but is due to increased erythropoiesis [47] that can be driven by the increased EPO levels. A few studies have also reported an increase in EPO after treatment with SGLT2is [22,23,48]. The specific mechanisms underlying the increased production of EPO due to SGLT2is are not entirely clear, but several potential pathways have been proposed.

(a) SGLT2is reduce glucose reabsorption in the renal tubules, potentially decreasing cellular stress and thereby improving renal EPO production [49]. This reduction in glucose reabsorption could lead to lowered energy consumption and increased oxygen availability in the kidneys, which might enhance EPO production [47,49]. (b) The increased workload on the distal tubules might lead to hypoxia at the corticomedullary junction, inducing hypoxia-inducible factor 1 (HIF1). The activation of HIF1 is known to enhance EPO production [50]. (c) SGLT2is may reduce the levels of hepcidin and ferritin, which would promote the mobilization of iron in the cytosol. Increased iron availability could trigger enhanced signaling through hypoxia-inducible factor 2 alpha (HIF2α), leading to increased EPO production [23,51]. (d) SGLT2is might upregulate SIRT1, a nutrient deprivation signaling pathway, which could also contribute to higher EPO production [52]. However, these mechanisms remain purely speculative, as the measurements of these markers have not been performed herein.

In this study we confirmed previous results regarding a decrease in ferritin levels and in transferrin saturation. This drop in ferritin levels was also seen in a recently published randomized control trial with heart failure patients treated with dapagliflozin after 3 months of treatment [53]. In another study it was shown that a 3-month treatment with dapagliflozin led to similar changes in hematocrit, TIBC, transferrin saturation and ferritin as in our study [23], and the authors proposed the reduced hepcidin levels as a contributory mechanism to the increased use of iron stores. A proteomics analysis of plasma of patients treated with empagliflozin also showed a decline in ferritin levels [54]. Last, according to another study, the increase in EPO levels per se drives the production of blood cells that use iron stores at a faster rate than they can be replaced and leads to reduced ferritin levels [55]. The decrease in parallel of ferritin and TSAT shows a signature of iron being consumed by erythropoiesis faster than it can be replenished rather than a reduction in inflammation where TSAT should have remained unchanged or increased.

NT-proBNP is a well-defined marker of heart failure [56]. It has been shown that in patients with heart failure with reduced ejection fraction a treatment with empagliflozin reduced the NT-proBNP levels and attenuated the risk for adverse heart failure or renal outcomes regardless of baseline NT-proBNP levels [57]. It has also been shown that NT-proBNP increases in the case of inflammation [37] and inflammatory diseases such as inflammatory arthritis [58]. In our study there was no statistically significant difference in the levels of NT-proBNP after SGLT2i treatment. This could be attributed to the fact that our patients had low NT-proBNP values at baseline (with the exception of four with values higher than 450 pg/mL), as they did not have clinically significant heart failure. However, a trend for decreased NT-proBNP levels was still detected with no statistical significance (Table 4 and Table 5). Similarly, the circulating levels of IL-6 were evaluated as an index of inflammation. Even though no statistically significant differences were detected, a trend toward lower IL-6 levels after SGLT2i treatment was evident. A recent meta-analysis of 18 studies showed an association between SGLT2i treatment and lower IL-6 levels [59], and another study showed a decrease in both NT-proBNP and IL-6 levels [60].

Another interesting aspect is the effect of SGLT2is on lipid metabolism and its association with the ameliorated cardiovascular outcomes. A number of studies showed a modest yet significant increase in total cholesterol, LDL-C, HDL-C and non-HDL-C [26] and a reduction in triglyceride levels after an SGLT2i treatment [61]. Despite the increase in LDL cholesterol, the SGLT2is have a cardioprotective effect. A randomized study with empagliflozin treatment of T2DM patients showed increased LDL cholesterol and LDL apolipoprotein B levels but no difference in calculated LDL particle size [62]. Another randomized study with dapagliflozin showed a decrease in small, dense low-density lipoprotein cholesterol and an increase in high-density lipoprotein 2-cholesterol, and the increased LDL was due to the increase in the less atherogenic lb LDL cholesterol [63]. LDL-C quantifies the cholesterol carried within LDL particles, whereas the LDL particle number reflects the count of circulating apoB-containing particles, and the two dissociate whenever particle composition changes. Since atherogenic risk is more closely determined by particle number than by cholesterol content, a rise in LDL-C occurring without a corresponding rise in particle number would denote cholesterol enrichment of existing particles rather than an increase in atherogenic particle burden. Our observation of the unchanged LDL particle number and particle diameter is compatible with this interpretation and aligns with previous reports that empagliflozin raised LDL cholesterol and LDL apolipoprotein B without altering the calculated LDL particle size [62] and that the LDL cholesterol increase with dapagliflozin was attributable predominantly to the less atherogenic large-buoyant subfraction, accompanied by a reduction in small, dense LDL cholesterol [63]. The mechanisms underlying these effects on lipids are not entirely clear. We emphasize, however, that our cohort was not powered to detect particle-number changes of the magnitude corresponding to the modest cholesterol shifts described in large trials, and the present findings should therefore be regarded as compatible with, rather than confirmatory of, this interpretation. An additional consideration is that all participants were receiving statin therapy, and the resulting reduction in apoB particle number may have attenuated any incremental effect of empagliflozin on lipoprotein composition [64].

In the present study we used an NMR-based assay to evaluate the effect of a 3-month SGLT2i treatment on cholesterol, triglyceride and particle numbers in all individual lipoprotein classes. To the best of our knowledge, this is the first time that such a thorough analysis was performed after an SGLT2i treatment. Moreover, the global plasma glycoproteins GlycA and GlycB were assessed for the first time using the same methodology. The concentrations of GlycA and GlycB can serve as novel biomarkers of inflammation [39,65]. Briefly, the NMR-based analysis did not reveal any differences in cholesterol or triglycerides in the individual lipoprotein classes (VLDL, LDL, IDL and HDL), in individual lipoprotein particle numbers (large, medium and small VLDL; large, medium and small LDL; large, medium and small HDL) or in the particle diameter of VLDL, LDL and HDL. No difference was seen in the concentration of glycoproteins GlycA, GlycB and GlycF or in the height/width ratios of GlycA and GlycB (markers of inflammatory status) before and after SGLT2i treatment.

Another study with a 12-week treatment with dapagliflozin did not observe any change in HDL cholesterol levels, HDL particle size, or the activity of enzymes mediating HDL anti-oxidative properties [66]. Last, it is important to note that the effect of SGLT2i on lipoproteins may be affected by whether patients were already taking statins or not. In this context, the addition of 10 mg of dapagliflozin to diabetics treated with rosuvastatin in patients with a well-controlled lipid profile did not affect total plasma cholesterol, LDL cholesterol, apoB, or triglyceride levels and caused only a modest increase in plasma HDL cholesterol [64]. In our study all the patients were already on statins, and thus it is possible that the effect of SGLT2is on lipid profiling was masked by the protective effect of statins.

One advantage of this study was that these patients were enrolled from a diabetes outpatient clinic, and empagliflozin was added as part of their regular treatment. Hence, the results of this study reflect a real-life experience with an SGLT2i. Another significant advantage of this study was the relative homogeneity of the population (Table 1 and Table 2) and the design, whereby each participant served as their own control. Sampling was conducted both before and after the treatment, allowing for a direct comparison of changes within individuals. Moreover, no other drugs besides the SGLT2i were altered during the study period. Nevertheless, this study has several limitations. Firstly, it is an observational cohort study, and it does not include a control arm, meaning patients not having received a placebo given the nature of the study. Thus, it was only possible to use each subject as a control of him/herself by comparing before- and after-treatment effects. Specifically, the SGLT2i treatment was introduced as an add-on for diabetes treatment; thus, the effects seen may be also the results of better glycemic control after initiation of the treatment. Moreover, one representative SGLT2i was used (empagliflozin), and thus the results can not necessarily reflect the effects of all drugs of this category. The analysis of lipoproteins was conducted on a separate, smaller cohort of patients who had received SGLT2i treatment for three months, making it relatively adequate for a large effect and inadequate for a moderate one. In contrast, the other measurements were assessed after a treatment duration of one month. Consequently, the two groups could not be combined for the purposes of analysis.

In conclusion, our study showed changes in iron metabolism, magnesium levels and inflammatory markers after empagliflozin treatment. The neutral effect of SGLT2i treatment on lipoprotein profiling suggests that the cardioprotective effects of these drugs may not be mediated through the modulation of lipoprotein species, even though this arm was underpowered to detect small or moderate changes. More research is warranted to delineate the molecular mechanisms underlying the pleiotropic effects of SGLT2is.

## 5. Conclusions

In this real-life cohort of T2D patients profiled before and after the addition of empagliflozin, the changes we observed did not distribute evenly across the parameters we examined: they clustered almost entirely within the erythropoietic–iron axis, while the inflammatory and lipoprotein parameters remained unchanged. Within one month, EPO, hemoglobin and hematocrit rose while ferritin and transferrin saturation fell—a pattern that is internally coherent and difficult to reconcile with hemoconcentration, since plasma-volume contraction would raise ferritin rather than lower it. Our findings therefore extend to a routine outpatient setting, which has been reported in selected trial populations, where the hemoglobin rise has been attributed to genuine erythropoiesis rather than dilution [47] and where comparable falls in ferritin and transferrin saturation have been documented with dapagliflozin over three months [23,53] and with empagliflozin by proteomic analysis [54]. Read together with the mediation analyses in which hemoglobin accounted for a substantial share of the cardiovascular benefit of these agents [17,46], the direction and timing of our observations position the erythropoietic response as an early and reproducible effect of SGLT2 inhibition rather than a late adaptation. Against this, the neutrality of the NMR-derived lipoprotein and glycoprotein profiles is informative in its own right: in a statin-treated population, we found no detectable remodeling of lipoprotein subclasses, particle numbers or particle size, nor of the glycoprotein markers GlycA and GlycB—suggesting that whatever cardioprotection these drugs confer in patients such as ours is unlikely to be transmitted through lipoprotein or systemic inflammatory remodeling, although the size of that cohort permits only large effects to be excluded.

## Figures and Tables

**Table 1:** Baseline characteristics of group G1 exposed to empagliflozin for 1 month (*n* = 42).

Age (Years) ± SD	53.2 ± 5.5
Gender	25 males (59.5%), 17 females (40.5%)
BMI (kg/m^2^) ± SD	31.5 ± 3.2
HbA1C (%) ± SD	7.5 ± 0.9
Creatinine (mg/dL)	0.99 ± 0.24

**Table 2:** Baseline characteristics of group G2 exposed to empagliflozin for 3 months (*n* = 17).

Age (Years) ± SD	50.4 ± 4.9
Gender	10 males (59%), 7 females (41%)
BMI (kg/m^2^) ± SD	30.6 ± 3.1
HbA1C (%) ± SD	7.7 ± 0.8
Creatinine (mg/dL)	0.88 ± 0.28

**Table 3:** Serum electrolytes, urinary glucose levels and urinary anion gap before and after 1-month treatment with empagliflozin.

	preSGLT2i	postSGLT2i	Mean Difference	*p*-Value
Sodium (mmol/L)	141.3 ± 12.61	144.3 ± 10.72	3 ± 15.88	0.2278
Potassium (mmol/L)	4.62 ± 0.56	4.71 ± 0.46	0.09 ± 0.57	0.3092
Magnesium (mg/dL)	1.93 ± 0.29	2.20 ± 0.25	0.264 ± 0.38	<0.0001
Creatinine (mg/dL)	0.99 ± 0.34	1.06 ± 0.39	0.076 ± 0.19	0.0134
Urine glucose (mg/dL)	7.04 ± 31.81	176.3 ± 109.6	169.3 ± 111.8	<0.0001
Urine anion gap	32.05 ± 30.22	29.67 ± 25.38	−2.39 ± 29.73	0.6191

**Table 4:** Serum levels of iron metabolism and inflammation markers after 1-month treatment with empagliflozin.

	preSGLT2i	postSGLT2i	Mean Difference	*p*-Value
Hct %	41.75 ± 3.59	43.48 ± 3.54	1.74 ± 2.078	<0.0001
Hgb (g/dL)	13.66 ± 1.37	14.11 ± 1.39	0.45 ± 0.78	0.0005
Ferritin (ng/mL)	158.6 ± 142.3	129.3 ± 124.6	−29.23 ± 51.97	0.001
Iron (μg/dL)	92.17 ± 39.51	83.86 ± 24.45	−8.31 ± 31.72	0.0972
TIBC (μg/dL)	352.6 ± 78.84	378.1 ± 83.9	25.48 ± 68.44	0.0237
Transferrin saturation (%)	26.61 ± 9.94	22.97 ± 7.47	−3.65 ± 7.22	0.0049
EPO (mU/mL)	15.46 ± 9.16	20.74 ± 13.88	5.28 ± 10.85	0.0030
IL-6 (pg/mL)	10.05 ± 15.71	7.55 ± 4.70	−2.5 ± 16.04	0.2909
NT-proBNP (pg/mL)	354.1 ± 1193	206.3 ± 428.4	−147.8 ± 895	0.2635

**Table 5:** Serum lipoprotein measurements after 3-month treatment with empagliflozin.

	preSGLT2i	postSGLT2i	Mean Difference	*p*-Value
VLDL cholesterol (mg/dL)	22.84 ±14.84	21.08 ± 11.34	−1.77 ± 7.85	0.55
IDL cholesterol (mg/dL)	16.67 ± 7.488	16.51 ± 6.313	−0.15 ± 6.17	0.92
LDL cholesterol (mg/dL)	91.68 ± 26.99	87.26 ± 19.00	−4.42 ± 23.47	0.35
HDL cholesterol (mg/dL)	47.31 ± 8.390	47.73 ± 8.723	0.43 ± 5.75	0.93
VLDL triglycerides (mg/dL)	95.75 ± 51.61	86.34 ± 46.13	−9.41 ± 27.52	0.31
IDL triglycerides (mg/dL)	15.22 ± 4.511	15.18 ± 3.177	−0.04 ± 3.54	0.96
LDL triglycerides (mg/dL)	19.20 ± 6.402	18.91 ± 4.351	−0.29 ± 5.61	0.82
HDL triglycerides (mg/dL)	16.38 ± 5.700	14.87 ± 2.754	−1.51 ± 3.99	0.33
VLDL particle number (nmol/L)	69.78 ± 39.51	63.33 ± 34.12	−6.45 ± 20.85	0.43
Large VLDL particle number (nmol/L)	1.699 ± 0.7743	1.546 ± 0.7383	−0.15 ± 0.45	0.17
Large VLDL particle %	2.559 ± 0.3200	2.502 ± 0.3071	−0.06 ± 0.36	0.72
Medium VLDL particle number (nmol/L)	7.141 ± 3.651	6.465 ± 3.657	−0.68 ± 2.99	0.22
Medium VLDL particle %	10.43 ± 2.007	10.40 ± 4.151	−0.03 ± 5.19	0.98
Small VLDL particle number (nmol/L)	60.94 ± 35.30	55.32 ± 30.81	−5.62 ± 19.78	0.43
Small VLDL particle %	87.01± 2.008	87.10 ± 4.031	0.08 ± 5.07	0.78
LDL particle number (nmol/L)	984.4 ± 268.5	937.9 ± 191.8	−46.49 ± 250	0.40
Large LDL particle number (nmol/L)	162.3 ± 34.62	155.9 ± 26.77	−6.39 ± 28.24	0.28
Large LDL particle %	16.69 ± 1.634	16.91 ± 2.676	0.23 ± 3.22	0.68
Medium LDL particle number (nmol/L)	283.4 ± 116.7	273.9 ± 84.60	−9.50 ± 99.34	0.78
Medium LDL particle %	28.07 ± 3.944	28.93 ± 5.641	0.86 ± 5.46	0.61
Small LDL particle number (nmol/L)	538.6 ± 127.4	508.0 ± 125.1	−30.61 ± 161.2	0.40
Small LDL particle %	55.25 ± 4.250	54.15 ± 6.887	−1.1 ± 7.15	0.52
HDL particle number (nmol/L)	23.00 ± 4.156	23.03 ± 4.043	0.03 ±3.4	0.93
Large HDL particle number (nmol/L)	0.2965 ± 0.05207	0.2794 ± 0.02657	−0.02 ± 0.05	0.22
Large HDL particle %	1.322 ± 0.2935	1.245 ± 0.2335	−0.08 ± 0.27	0.32
Medium HDL particle number (nmol/L)	11.19 ± 1.669	10.78 ± 1.666	−0.41 ±2.09	0.36
Medium HDL particle %	49.74 ± 9.166	47.79 ± 9.592	−1.95 ±10.03	0.20
Small HDL particle number (nmol/L)	11.51± 3.805	11.97 ± 3.976	0.46 ±3.85	0.58
Small HDL particle %	48.94 ± 9.382	50.97 ± 9.767	2.03 ± 10.23	0.21
GlycB (μmol/L)	433.2 ± 60.75	427.9 ± 51.42	−5.32 ± 61.04	0.93
GlycF (μmol/L)	304.3 ± 67.17	290.9 ± 58.83	−13.39 ± 54.8	0.46
GlycA (μmol/L)	824.2 ± 184.5	818.3 ± 175.2	−5.95 ± 129.8	0.93
H/W Glyc-B	5.454 ± 0.7670	5.383± 0.6543	−0.07 ± 0.78	0.89
H/W Glyc-A	18.64 ± 3.134	18.67± 2.918	0.03 ± 2.59	0.97
IL-6 (pg/mL)	10.14 ± 18.87	8.58 ± 10.31	−1.56 ± 9.08	0.96
NT-proBNP (pg/mL)	145.2 ± 216.2	92.27 ± 128.9	−52.97 ± 125.2	0.22

## Data Availability

The data presented in this study are available on request from the corresponding author due to privacy reasons.

## Abbreviations

The following abbreviations are used in this manuscript:

apoB	Apolipoprotein B
CKD	Chronic kidney disease
Cl	Chloride
ECL	Electrochemiluminescence
eGFR	Estimated glomerular filtration rate
EPO	Erythropoietin
GlycA	Glycoprotein A
GlycB	Glycoprotein B
GlycF	Glycoprotein F
HbA1c	Glycated hemoglobin (hemoglobin A1c)
HDL	High-density lipoprotein
HDL-C	High-density lipoprotein cholesterol
HFpEF	Heart failure with preserved ejection fraction
HFrEF	Heart failure with reduced ejection fraction
HIF1	Hypoxia-inducible factor 1
HIF2α	Hypoxia-inducible factor 2 alpha
H/W	Height/width (ratio)
^1^H-NMR	Proton nuclear magnetic resonance
IDL	Intermediate-density lipoprotein
IL-6	Interleukin-6
K	Potassium
LDL	Low-density lipoprotein
LDL-C	Low-density lipoprotein cholesterol
Na	Sodium
NMR	Nuclear magnetic resonance
non-HDL-C	Non-high-density lipoprotein cholesterol
NT-proBNP	N-terminal pro–B-type natriuretic peptide
SGLT2	Sodium–glucose cotransporter 2
SGLT2i	Sodium–glucose cotransporter 2 inhibitor(s)
SIRT1	Sirtuin 1
T2DM	Type 2 diabetes mellitus
TIBC	Total iron binding capacity
VLDL	Very-low-density lipoprotein
